# Describing chain-like assembly of ethoxygroup-functionalized organic molecules on Au(111) using high-throughput simulations

**DOI:** 10.1038/s41598-021-93724-5

**Published:** 2021-07-19

**Authors:** Lokamani, Jeffrey Kelling, Robin Ohmann, Jörg Meyer, Tim Kühne, Gianaurelio Cuniberti, Jannic Wolf, Guido Juckeland, Thomas Huhn, Peter Zahn, Francesca Moresco, Sibylle Gemming

**Affiliations:** 1 Department of Information Services and Computing, Helmholtz-Zentrum Dresden-Rossendorf (HZDR), Bautzner Landstraße 400, 01328 Dresden, Germany; 2 Institute of Ion Beam Physics and Materials Research, Helmholtz-Zentrum Dresden-Rossendorf (HZDR), Bautzner Landstraße 400, 01328 Dresden, Germany; 3 Institute for Materials Science, Technische Universität Dresden, 01062 Dresden, Germany; 4 Department of Physics, Universität Siegen, Walter-Flex-Straße 3, 57072 Siegen, Germany; 5 Center for Advancing Electronics Dresden, Technische Universität Dresden, 01062 Dresden, Germany; 6 Department of Chemistry, Universität Konstanz, 78457 Konstanz, Germany; 7 Institute of Physics, Technische Universität Chemnitz, 09107 Chemnitz, Germany

**Keywords:** Molecular self-assembly, Molecular electronics

## Abstract

Due to the low corrugation of the Au(111) surface, 1,4-bis(phenylethynyl)-2,5-bis(ethoxy)benzene (PEEB) molecules can form quasi interlocked lateral patterns, which are observed in scanning tunneling microscopy experiments at low temperatures. We demonstrate a multi-dimensional clustering approach to quantify the anisotropic pair-wise interaction of molecules and explain these patterns. We perform high-throughput calculations to evaluate an energy function, which incorporates the adsorption energy of single PEEB molecules on the metal surface and the intermolecular interaction energy of a pair of PEEB molecules. The analysis of the energy function reveals, that, depending on coverage density, specific types of pattern are preferred which can potentially be exploited to form one-dimensional molecular wires on Au(111).

## Introduction

Molecules can form interlocked lateral patterns after being deposited on metal surfaces. The process of pattern formation may depend on various factors, in particular the competition between molecule-substrate and intermolecular interaction. The formation of interlocked lateral patterns has been investigated recently using low temperature scanning tunneling microscopy (STM)^1^, where laterally interlocked chains were grown epitaxially from inorganic ionic cyanides. In case of organic molecules with $\pi$-conjugated backbones adsorbed on Au(111) substrate, e.g. phenylenes and phenyleneethynylenes^2^, the pattern-formation is mediated by a relatively weak van der Waals force and the direct interactions of the functional groups between the molecules. The interaction between organic molecules and inorganic substrate at the atomic level and interaction mediated pattern formation plays a crucial role in device characteristics^3^. It is essential to understand the underlying mechanism of such interactions in order to design and fabricate new molecules with enhanced functionality and stability for organic electronic devices^4^.

In this article, we present the results of a combined theoretical and experimental study of elongated organic 1,4-bis(phenylethynyl)-2,5-bis(ethoxy)benzene (PEEB) molecules deposited on Au(111), which can also form quasi-interlocked lateral patterns on Au(111), as revealed through investigation by low temperature (STM). In order to investigate whether those patterns depend specifically on the molecular structure of PEEB, we scan the space of possible adsorption geometries by high-throughput calculations^5,6^ using density-functional-based tight-binding plus (DFTB+)^7^. The present study follows the choice of high-symmetry adsorption sites for atoms and diatomic molecules in^8^, but additionally employs the symmetry of the substantially larger molecules to define an irreducible wedge in configuration space and sample only that sector. We thus explore an energy landscape which describes the adsorption of single PEEB molecules on the Au(111) surface and the interaction within a molecular pair.

High throughput methods have recently gained increasing interest, because they allow the automated search for thermodynamically stable structural and magnetic phases^9–15^ and also serve to determine average numerical parameters for classical and quantum mechanical simulation approaches^16–19^. Consequently, high-throughput methods to screen compounds for desirable physical properties of interest have become the major driving force behind numerous data driven materials prediction methods^20–24^. Rather than applying the aforementioned methods to screen a range of compounds, we employ a homogeneous sampling strategy to explore the configurational energy landscape of a single system with the objective of describing intricate steric and electronic interactions of molecular pairs to understand the building blocks of the experimentally observed pattern formation. We demonstrate a visual approach of decomposing the multiple dimensions of the energy landscape to track down all prominent pair formations.

## Results

### Experimental results

**Figure 1:**
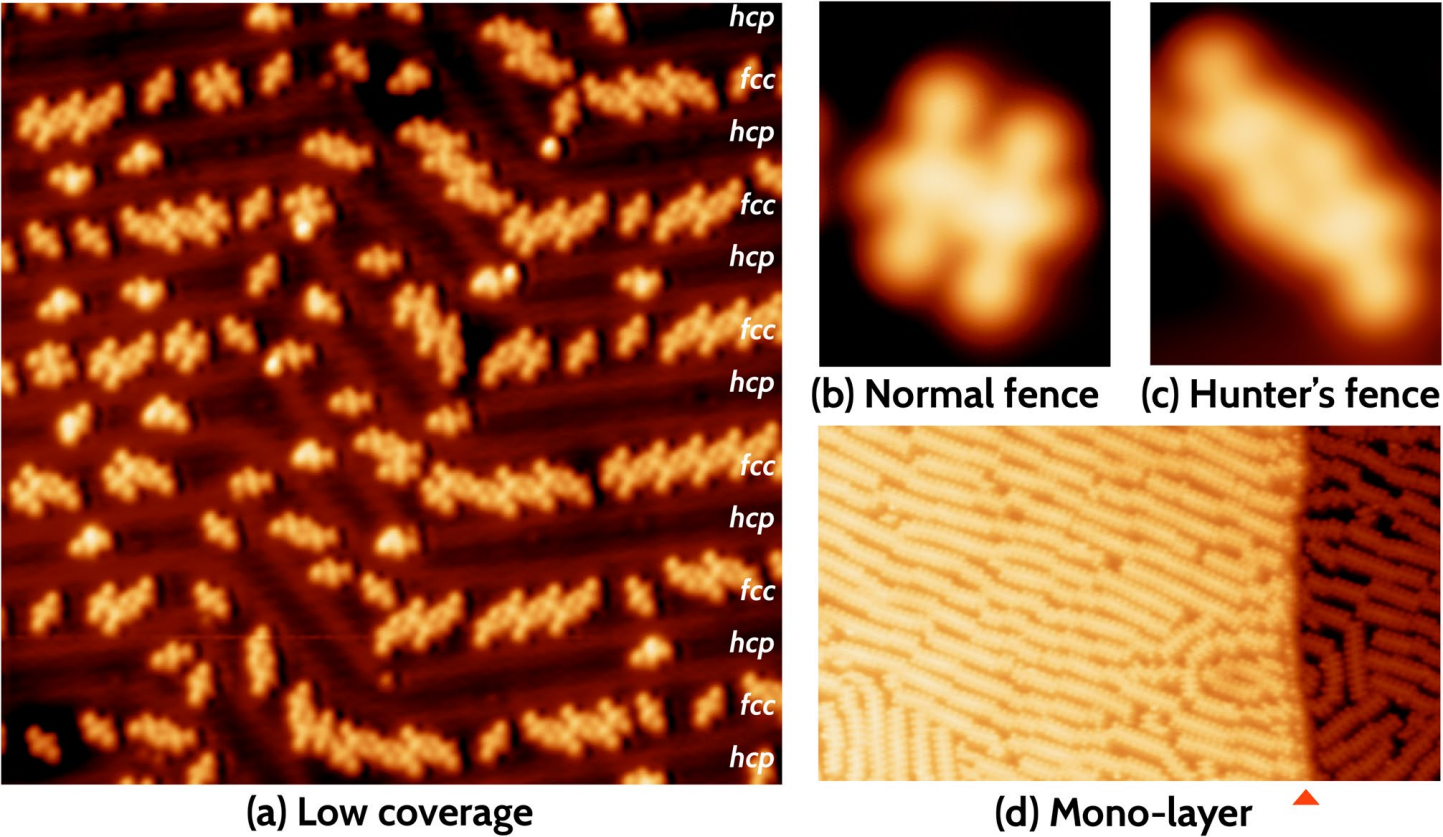
(**a**) STM images of PEEB adsorbed on Au(111) at low coverage (Image size: $40\times 40\ \mathrm{nm}^{2}$, $V_{{{\text{appl}}}} = - 0.5\;{\text{V}},I = 50\;{\text{ pA}}$). At low coverage PEEB molecules adsorb predominantly on fcc-regions. Two prominent clustering patterns are denoted as (**b**) *Normal fence* (image size: $2.8 \times 3.5\;{\text{nm}}^{2}$, $V_{{{\text{appl}}}} = - 0.1\;V,\;I = 50\;{\text{pA}}$) and (**c**) *Hunter’s fence* (image size: $2.8 \times 3.5\;{\text{nm}}^{2}$, $V_{\rm {appl}} = {-1}\ {\rm{V}}, I = {50} \text { pA}$) (**d**) On increasing the coverage of the PEEB molecules, the *normal fence*-like clustering into long one-dimensional rows is observed only. The coverage of the one-dimensional rows extends until the step edges. Step edge shown using red triangle (image size: $70\times {40}\ {\mathrm{nm}^{2}}$, $V_{{{\text{appl}}}} = - 1\;{\text{V}},\;I = 50\;{\text{pA}}$). Note that, the atomic resolution of the substrate is not possible, while imaging adsorbed molecules because different tunneling parameters are employed. The exact adsorption position can therefore only be determined by density functional theory (DFT) calculations.

In the STM experiments, various kinds of molecular clustering are observed at different degrees of coverage for PEEB molecules on Au(111) at low temperatures. At a low degree of coverage, the PEEB molecules cluster predominantely into two *fence-like* structures: *normal fence* and *hunter’s fence*, as shown in Fig. 1b,c, respectively. As the concentration of the molecules and with that the degree of coverage on the gold substrate is increased, only rows formed in a normal-fenced pattern are observed (see Fig. 1d). At low coverage, the adsorption occurs preferentially on the fcc-region of the reconstructed Au(111) surface.

### Single PEEB molecule: adsorption on Au(111)

A combined theoretical and experimental study of PEEB molecules deposited on Au(111) is presented in a recent publication^25^. The electronic and geometric structure of a single PEEB molecule on Au(111) surface is investigated using low-temperature STM. The density-functional-based tight-binding (DFTB)^7^ simulations of the geometrical configuration, density of states (DOS), and charge-reordering and charge-transfer suggest, that PEEB molecules are physisorbed on Au(111). The work of adhesion equals $0.31\;{\text{Jm}}^{{ - 2}}$, implying a non-reactive wetting of Au(111) by PEEB molecules. Our analysis reveals not only a negligible change of geometrical structure upon adsorption but also negligible absolute charge transfer per atom of mere $0.003|{\text{e}}|$ to the substrate. The total charge transfer between the molecule and the substrate equals $0.14|{\text{e}}|$, which excludes the possibility of intermolecular Coulomb interaction. Furthermore, the charge re-distribution for the molecule occurs predominantly along the side chains (SCs) and the outer phenyl ring (OPR), which is not perturbed significantly upon adsorption. An overall dipole moment is prohibited by the presence of an inversion center. Thus, a possible clustering of PEEB molecules would be driven by interactions between partial-charge centers.

### High-throughput calculations: clustering

In order to explain the clustering of PEEB molecules on Au(111) we propose an energy function incorporating two concurrent yet independent processes: (a) a Surface Binding Energy, which describes the adsorption of single PEEB molecules on Au(111) on a chosen site and (b) a Planar Clustering Energy, which takes into consideration the intermolecular interactions solely between two isolated PEEB molecules. We define the sum of both energies as total energy $E_{{{\text{tot}}}}$.

**Figure 2:**
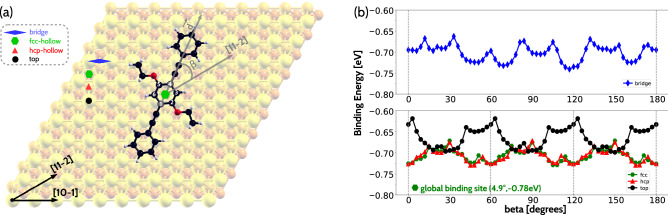
(**a**) Sampling shown for the central phenyl ring placed over an fcc-hollow site: $\beta$ denotes the in-plane rotational angle of MB to the [11-2] direction. The vector $\vec {d}$ defining the MB-direction is spanned between the carbon atoms 1 and 4 of the central phenyl ring. $\beta$ has the value $0^{\circ }$, if the molecular backbone is oriented parallel to the [11-2] direction. The bridge-, fcc-, hcp-, top-sites are shown using blue skewed diamond, green hexagon, red triangle and black circle, respectively. There is an atom directly beneath the third (second) layer below the fcc (hcp) hollow site, respectively. (**b**) Binding energies: The configurational spaces bridge-ms, fcc-ms, hcp-ms, and top-ms are denoted using blue, green, red, and black curves, respectively. fcc-ms, hcp-ms and top-ms curves reflect a three-to-six-fold rotational symmetry, whereas bridge-ms does not show any additional symmetry between $0^{\circ }$ and $180^{\circ }$, as expected.

We sample the configurational space in search for the most likely interlocking patterns in two steps: At first, we determine the energy landscape of the binding process of a single molecule on the surface. For that, we place an isolated PEEB molecule over Au(111) with the central phenyl ring (CPR) aligned over a high-symmetry site at the Au surface. The in-plane rotation angle $\beta$ of the start configuration is choosen as $0^{\circ }$, when the molecular backbone (MB) is parallel to the [11-2] direction of the Au(111) surface, see Figure 2(a). Subsequently, $\beta$ is increased to $180^{\circ }$ in steps of $3^{\circ }$, while keeping the center of the CPR aligned to the chosen adsorption site. As the molecule exhibits an inversion center, only angles of at most $180^{\circ }$ need to be scanned, thus the number of configurations considered are 61. Lower number of configurations are needed at sites with higher local symmetry of Au(111). The average distance between the carbon atoms and the topmost gold layer is set to 4 Å in the start configuration. The so-generated configurations are relaxed with the following geometrical constraints: a) the gold atoms of the substrate are kept fixed, b) the CPR is allowed to relax only in the z-direction and c) the remaining atoms of the molecule are relaxed without any constraints. This procedure is repeated for the inversion center in the middle of the central phenyl ring placed over an fcc-hollow, an hcp-hollow, a top and a bridge position. Four subgroups on fcc, hcp, top and bridge sites span the configurational space: fcc-ms, hcp-ms, top-ms and bridge-ms, where ms denotes molecular-substrate system. The constraint (b) ensures, that the final configurations are unique and the sampling of the configurational space is unbiased^26–29^. The choice of the supercell and the spurious interactions caused by the periodic boundary conditions are discussed in the supplemental information (SI).

The surface binding energies for the configurations are shown in Fig. 2b. As expected, the green, red and black curves corresponding to the binding energies of the fcc-, hcp- and top-configurational space resemble very closely a 6-fold symmetry. This is due to the fact, that a particular configuration and its counterpart rotated by $60^{\circ }$ about the CPR in the fcc-ms, hcp-ms and top-ms configurations are geometrically equivalent and have essentially the same total energies. On the other hand, the blue curve corresponding to the binding energy of the bridge-configurational space does not show any rotational symmetry within the scanned angle range. This reflects the reduced local symmetry of the bridge position. Within the bridge-ms configurational space, a particular starting configuration and its counterpart rotated by $180^{\circ }$ about the CPR are geometrically equivalent and have the same binding energy. The deviations in binding energies from the ideal symmetric case are visible at $90^{\circ }$ as compared to at angles of $30^{\circ }$ and $150^{\circ }$ for the green, red and black curves. These deviations are caused by the difference in the strength of interaction between the molecule and its periodic images attributed to the alignment of the MB along the short and long diagonal of the supercell. These deviations depend on the form and dimension of the periodic supercell which induce a symmetry-break.

**Figure 3:**
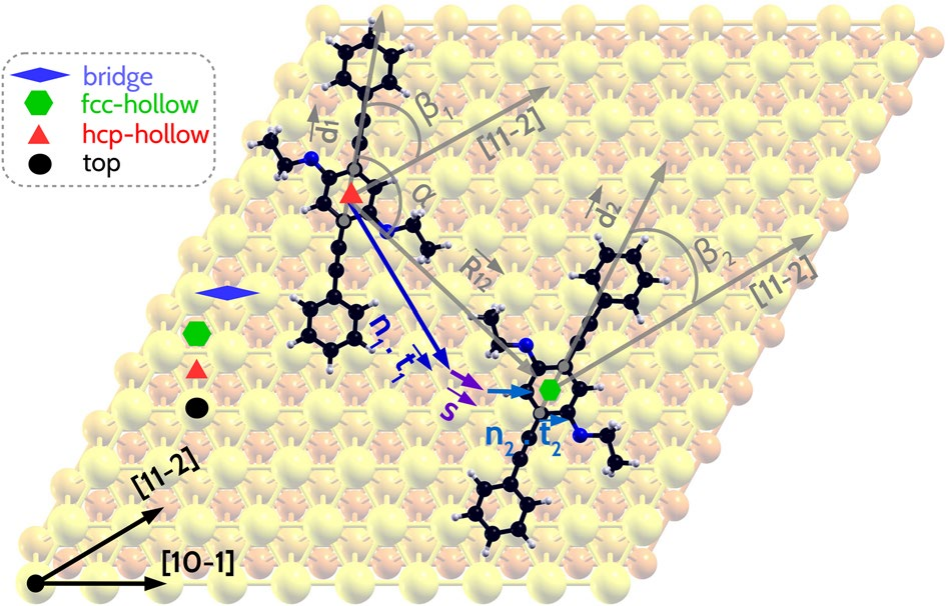
Formation of a molecular pair from the configurational space subgroup [hcp-m|fcc-m]: CPR of the first molecule is on a hcp-hollow site, of the second molecule on a fcc-hollow site. The in-plane rotation of the MB of the first molecule w.r.t [11-2] is $\beta _{1} =$ $54^{\circ }$, and of the second molecule $\beta _{2} =$ $27^{\circ }$. The total distance between the centers of mass of the molecules corresponds to $3\overrightarrow{t_{1}}$ + $1\overrightarrow{t_{2}}$ + $\vec {s}$. The angle $\alpha$, which is formed between the direction vector $\overrightarrow{d_{1}}$ of the first molecule and the vector $\overrightarrow{R_{12}}$ connecting the centers of mass of both the molecules amounts to $122.4^{\circ }$.

Next, in search for the most likely interlocking patterns as the second step, we utilize the single molecule configurations fcc-ms, hcp-ms, top-ms and bridge-ms to sample the configurational space of the clustering of molecule pairs to form interlocking patterns. The geometries of the molecules are extracted from the single molecule configurations excluding the gold surface to form pure molecular configurational space subgroups: fcc-m, hcp-m, top-m and bridge-m. A new set of configurations of molecular pairs is formed by performing the following steps: (1) a particular configuration is chosen from fcc-m, hcp-m, top-m or bridge-m, (2) another configuration is chosen from fcc-m, hcp-m, top-m or bridge-m, 3) the second configuration is translated w.r.t. the first molecule in units of the lattice vectors $\overrightarrow{t_{1}} = [ 1, \bar{1}, 0 ]$ and $\overrightarrow{t_{2}} = [ 1, 0, \bar{1} ]$. Steps 1 and 2 ensure that all pairs of in-plane rotations of the MBs relative to the [11-2] surface direction are taken into account. Translations in units of the lattice vectors ensure that the second molecule preserves the geometrical invariance w.r.t. the chosen adsorption site for the inversion center of the PEEB molecule. These steps are repeated for all possible pairs of the pure molecular configurational subgroups: fcc-m, hcp-m, top-m and bridge-m. Clusters generated from identical configurational subgroups do not pose a binding site mismatch of the CPR, since both of the CPRs are centered on identical fcc-, hcp-hollow, top or bridge sites, respectively. In order to account for a binding site mismatch of the CPRs, which arises while generating a cluster of molecular pairs from different configurational subgroups e.g. [hcp-m|fcc-m], an additional shift vector $\vec {s}$ is applied to the translation of the second molecule. The shift vector in this case would be $\vec {s} = \frac{1}{3} [ \overrightarrow{t_{1}} + \overrightarrow{t_{2}} ]$. The procedure is schematically shown in Fig. 3.

**Figure 4:**
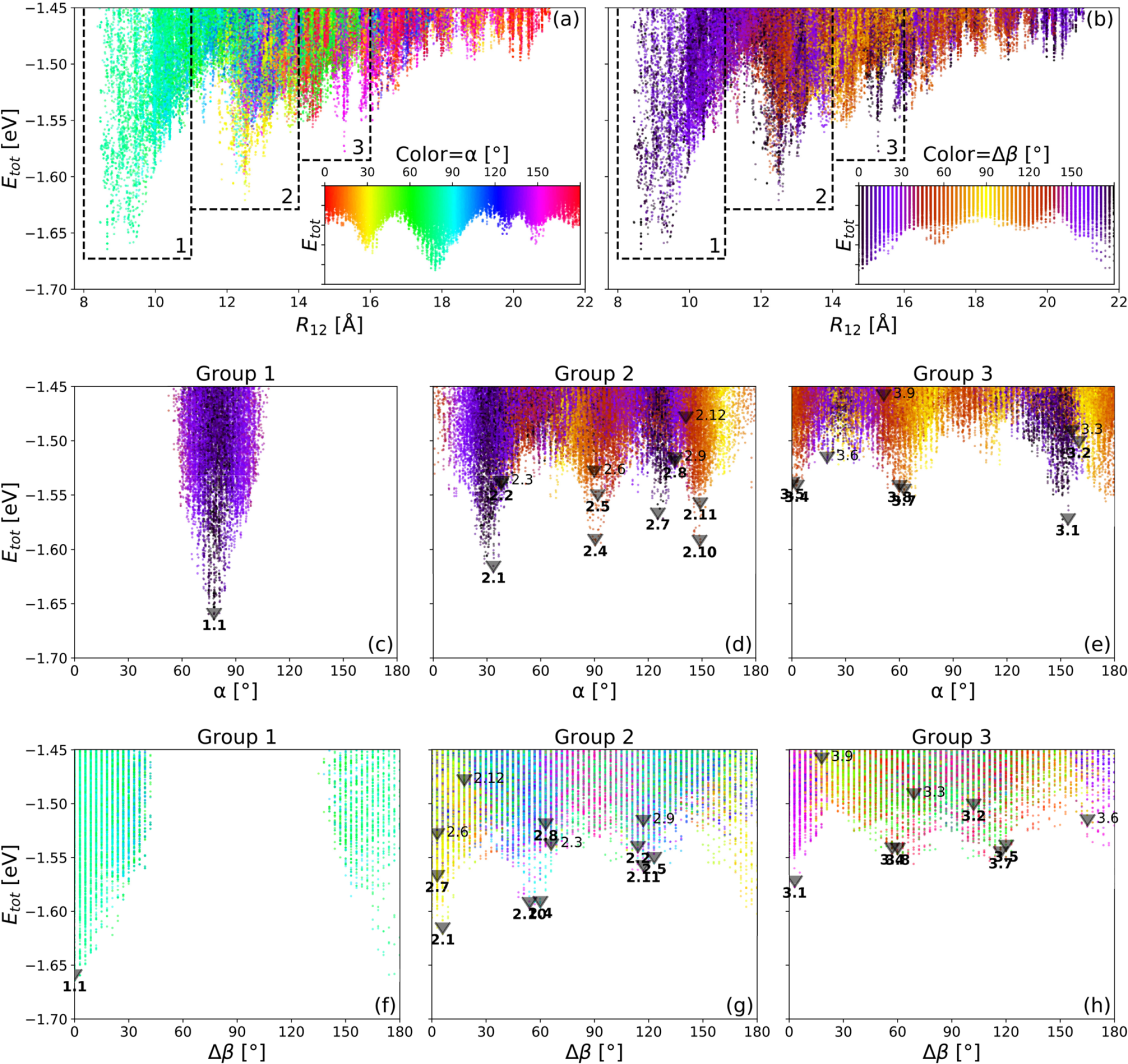
(**a**,**b**): Total energy $E_{{{\text{tot}}}}$ for pair configurations over distance ${R_{12}}$. The color maps show the position of one molecule relative to the other described by the angle $\alpha$ enclosed by one molecular backbone and the connection line, and the relative orientation angle $\Delta \beta$ between the molecules, respectively. The insets show the colorscales for the angles as $E_{{{\text{tot}}}} (\alpha )$ and $E_{{{\text{tot}}}} (\Delta \beta )$ plots. The insets reflect intrinsic symmetries of the system which are invariance against a rotation by $180^{\circ }$ in $\alpha$, panel (a), and mirror symmetry around $\Delta \beta =0^{\circ }$ and $90^{\circ }$, panel (**b**). The marked *y*-values in the insets are the same as in the main figure. Dashed frames mark the regions around the main peaks selected as distance groups for further analysis. (**c**-**e**): Total energy $E_{{{\text{tot}}}}$ for pair configurations over $\alpha$. Each panel shows configurations from one distance group. The labeled triangles indicate the positions of sampled configurations for each visible peak. The colors correspond to the relative orientation of the molecules by angle $\Delta \beta$, see panel (**b**) for the color key. (**f**-**h**): Total energy $E_{{{\text{tot}}}}$ for pair configurations over the relative orientation angle $\Delta \beta$ of the molecular backbones. Each panel shows configurations from one distance group only. The labeled triangles indicate the positions of sampled configurations for each visible peak. The colors correspond to the angle $\alpha$, see panel (**a**) for the color key.

This procedure is applied for all possible unique pair combinations (in total 10). The sampled configuration space for molecule pairs (MP-CS) is spanned by (a) a shift vector $\overrightarrow{R_{12}}$, which is spanned between the centers of mass of the molecules, (b) the difference between the angle of in-plane rotation of the individual molecules w.r.t. the [11-2] direction $\Delta \beta = \beta _1 - \beta _2$, and (c) the angle $\alpha$, which is defined as the angle between the following two vectors: (1) the directional vector $\overrightarrow{d_1}$ of the first molecule and (2) the vector spanned between the center of mass of the first molecule and the center of mass of the second molecule $\overrightarrow{R_{12}}$, shown in Fig. 3. The choice of the supercell is discussed in the SI (see Fig. S2).

### Analysis of molecule pair configurations

Figure 4a,b show the total energy over distance $R_{12}$ for all configurations. This reveals three local minima which are enclosed by the intervals numbered 1, 2 and 3, indicating corresponding groups of configurations: group 1 ($8 \r{A}  <R_{12} <11 \r{A}$), group 2 ($11 \r{A} < R_{12}  <14 \r{A}$) and group 3 ($14 \r{A} < R_{12} < 16 \r{A}$). Panel (a), colored according to $\alpha$, shows, that each of these three peaks is dominated by configurations with distinct relative alignments at angles $\alpha \approx 80^{\circ }$ for group 1, $30^{\circ }$ for group 2 and $150^{\circ }$ for group 3 w.r.t. the connection vector $\overrightarrow{R_{12}}$. The inset of panel (a) visualizes those three major orientations and indicates the presence of further weakly preferable configurations with $\alpha \approx 0^{\circ }$ and $130^{\circ }$, occurring for distances of $16 \r{A}$ and beyond. Panel (b) indicates the relative in-plane rotation $\Delta \beta$ of the two molecules depicted using color. The inset indicates that the relative angles $\Delta \beta =0^{\circ }$ and $180^{\circ }$ (parallel configurations) have a clear prevalence over the other angles. Given the elongation of the MB and its high aspect ratio of about 4:1, this finding reflects the geometrical constraints when adsorbing PEEB molecules on surfaces with 1D-dominated reconstructions such as the herringbone structure of Au(111).

Figure 4c-h decompose the identified groups further by presenting the energy over $\alpha$ in panels (c) to (e), and over $\Delta \beta$ in panels (f) to (h). For each distance group separately, we defined intervals around the pronounced $E_{{{\text{tot}}}} (\alpha )$-peaks and $E_{{{\text{tot}}}} (\Delta \beta )$-peaks for our interval outer product ansatz. The resulting minima of all interval products are labelled and marked. The two lowest minima out of each $E_{{{\text{tot}}}} (\alpha )$-peak are highlighted by bold font, the corresponding molecular-pair geometries are displayed in the supplemental material.

**Figure 5:**
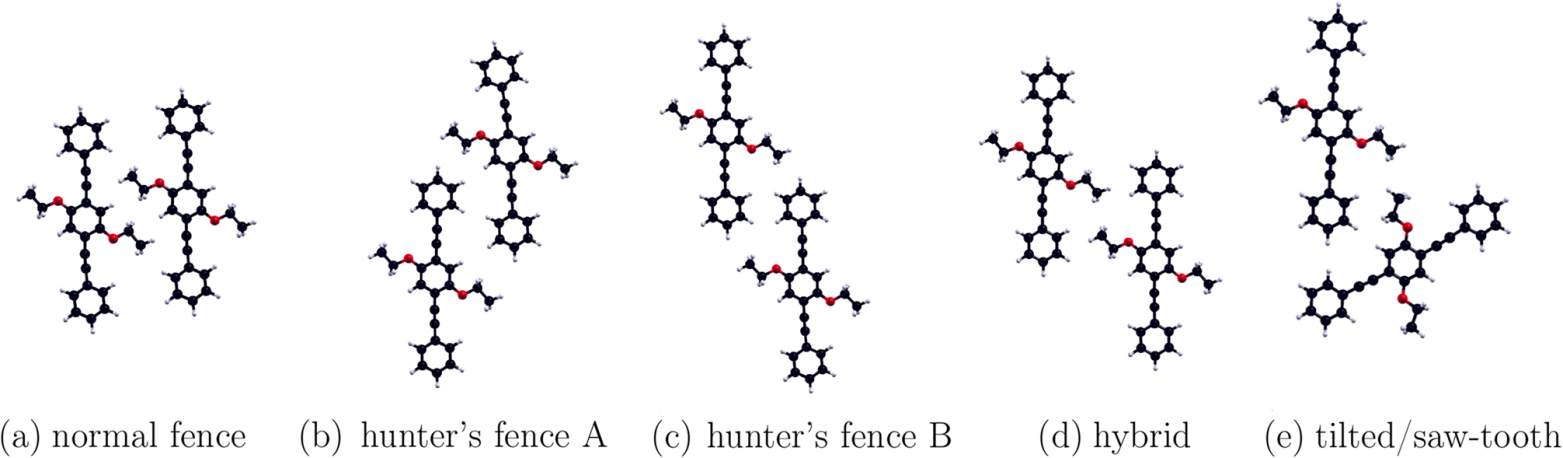
Prototypes for energetically most favorable pair configurations (**a**) *normal fence* 1.1 (**b**) *hunter’s fence A* 2.1 (**c**) *hunter’s fence B* 3.1 (**d**) *hybrid* 2.7 and (**e**) *saw-tooth* 2.4.

In Group 1, there is only one symmetrically unique minimum state, labelled 1.1, representing a configuration in which the molecules are parallel and only slightly offset ($\alpha \approx 80^{^\circ }$) at a distance of about $9 \r{A}$. We denote this configuration *normal fence*. A prototype is shown in Fig. 5a.

In Group 2, corresponding to larger distances of about $12.5 \r{A}$ between the centers, the *normal fence* configuration is no longer favorable. The minimum energy configuration, although still a parallel one, is strongly offset at $\alpha \approx {33.7}^{\circ }$ as indicated by the label 2.1. This configuration is shown as prototype in Fig. 5b and we denote it as *hunter’s fence A*. The runner-up configurations 2.2 and 2.3 could be considered marginal as they are close to the energy ($\approx - 1.55\;{\text{eV}}$) above which no peaks were considered. Another much less stable parallel configuration labelled 2.7 belongs to the peak with a principal angle $\alpha$ of about ${125.4}^{\circ }$. Because this angle is between that of the *normal* and *hunter’s fence* configurations we label it *hybrid*. A prototype of this is shown in Fig. 5d. Figure 4g shows subsequently energetically favorable non-parallel ($\Delta \beta \ne 0^{\circ }$, $180^{\circ }$) configurations, representing energy minima in the peaks marked 2.4 and 2.10, the runner-up configurations here, belong to a symmetrically equivalent peak at $\Delta \beta \approx 60^{\circ }$, $120^{\circ }$. The prototype 2.4 of this configuration, which we denote *saw-tooth*, is shown in Fig. 5e. The remaining second runner-ups are not of interest because their total energy is clearly above the base of their respective peaks.

In Group 3, we restrict to a single relevant peak with the prototype 3.1. It is similar to *hunter’s fence A* (see Fig. 5b), except that with an offset of $150^{\circ }$ in $\alpha$, the phenyl heads dock at the acute angle between arms and backbone of the other molecule, necessitating the larger distance. Due to this similarity, we denote this configuration *hunter’s fence B* shown in Fig. 5c. These two configurations are complementary in that, if the molecules were to densely cover a flat surface with *hunter’s fence A* chains, the perpendicular coordination would have to be *hunter’s fence B*.

All marked/selected configurations in Fig. 4 are shown explicitly in SI section II.

### Discussion: clustering models

The STM images reveal that the molecules adsorb preferentially on the fcc-domains of the herringbone reconstruction of the Au(111) surface. The preference of organic molecules for the fcc-domains on Au(111) has previously been reported in the literature^30^. Although no specific orientation of single molecules adsorbed on Au(111) is discernible, the molecules group together in two different configurations to form molecular clusters and chains (see Fig. 1). In the first configuration (Fig. 1b), the molecules group together in such a way that the side chains lie next to each other to form *normal-fence*-like structures. In the second configuration (Fig. 1c), the molecules group together with the side chains and the outer phenyl rings lying next to each other to form *hunter’s-fence*-like structures. At higher coverage the monolayer consists of the *normal-fence*-like structures only.

The analysis of the energy function supports the correlation of the adsorption pattern with the adsorbate density. If the distance between the central phenyl rings of adjacent molecules is between 8 Å and 11 Å, only *normal-fence*-like structures with mostly parallel molecules are preferred, as evident from the clustering analysis of group 1 (see Section III). This is in agreement with the STM observations of monolayers of PEEB molecules on Au(111), where the distance between the central phenyl rings of the molecules is minimum. The monolayers are comprised of rows of *normal-fence*-like structures as shown in Fig. 1d. For lower coverage of PEEB molecules on Au(111) and as the distance between the central phenyl rings of molecules increases, the *hunter’s-fence*-like and *hybrid* structures occur, in addition to the *normal-fence*-like structures. These characteristics of the groups 2 and 3 are in accordance with the STM observations of *normal-fence*-like and *hunter’s-fence*-like clusters at lower concentration as shown in Fig. 1a. Our calculations predict two forms of *hunter’s-fence*-like structures: *hunter’s fence A* and *hunter’s fence B*. Furthermore, our calculations suggest that *hunter’s fence A* is energetically more favorable than *hunter’s fence B*. Given the sparcity of experimental statistical data on the orientation and proximity of individual molecules to surrounding molecules, a similar distinction of *hunter’s-fence*-like structures in STM images is not viable.

Note that, Au adatoms can play an important role towards the formation of metalorganic structures on surface. Although, in most cases such Au adatoms are not visible in STM images^31–33^, their presence is however demonstrated by DFT calculations and simulated imaging. In the present case, the theory reproduces the cluster formation by interactions between the centers of partial charge centers on the PEEB molecule without including and Au adatoms.

In summary, the *high-throughput* calculations and the evaluation of the one- and two-particle terms of the *energy function* $E_{{{\text{tot}}}}$ attributed to the adsorption and clustering processes, elucidate the different principles involved in pattern formation of PEEB on Au(111) at various degrees of coverage.

## Methods

### Experimental setup

PEEB molecules were studied on the Au(111) surface with a low temperature STM at $5\;{\text{K}}$ under ultrahigh vacuum conditions ($<10^{-10} \ \mathrm{mbar}$). The gold single crystal surface was cleaned by repeated cycles of Ne ion sputtering and subsequent annealing at ${720}\ \mathrm{K}$. The molecules were deposited from a Knudsen cell at 420 K onto the Au(111) substrate which was kept at room temperature for about 10min. After the deposition, which takes place in a dedicated preparation chamber, the sample is cooled down with liquid nitrogen to about 85 K and then transferred into the STM chamber, without disrupting the vaccum conditions. The time needed to cool down the sample to 5 K is of the order of 10 h in the present setup. A bias voltage was applied to the sample, while the tip was grounded.

### Electronic structure methods

We performed self-consistent density-functional-based tight-binding simulations for geometric and electronic structural properties as implemented in the program package DFTB+^7^. The parameter set “auorg-1-1” has been utilized in all our calculations^34^, which is an extension of the “mio-1-1”^35^ parameter set to include gold. The “mio-1-1” set has been developed for organic molecules including O, N, C, H, and S atoms and works well for conformational energies and geometries of H-bonded systems^36,37^. The maximum angular momenta included in our calculations are: H:s, C:p, N:p, O:p and Au:d. In case of an isolated molecule and the geometries with gold substrate, the unit cell was spanned using following lattice vectors: $a_1 = [ 28.85 \r{A}, 0, 0 ]$, $a_2 = [ 14.43 \r{A}, 24.985 \r{A}, 0 ]$ and $a_3=[ 0, 0, 25 \r{A}]$. The Au(111) substrate was modelled using three layers ($10\times 10\times 3$)^3,31^, which were kept fixed (see Fig. S1 in the supplements). In case of pairs of molecules, the unit cell was doubled in both the $a_1$ and the $a_2$ directions. Periodic boundary conditions were used for all calculations. The relaxations were performed until the maximum force components reduced to 0.0001 eV/Å. All structural relaxation calculations were performed using the conjugate gradient algorithm including a universal force field extension for dispersion corrections as implemented in DFTB+^38^.

### Determining minimum configurations

We explore the energy landscape formed by adsorbed molecule pairs employing an intuitive graphical approach, which non the less guarantees, that all significant local minima, i.e. relevant pair configurations, are located regardless of the dimensionality of the problem. An absorbed pair can be characterized by three quantities, which we will define later: distance, relative position and relative orientation of the two molecules, giving rise to a hypersurface. Plotting total energy over each quantity separately will, in general, only reveal a subset of all significant local minima as pronounced peaks. Intervals enclosing a single peak each constitute regions of interest in each dimension. These intervals are defined to enclose the major part of the protrusion of the inverted peaks that are distinctly visible. Taking an outer product of the sets of intervals of all dimensions produces a super set of product intervals which encloses all significant local minima. The minimum energy configuration in each of these product intervals is either a local minimum configuration represented or hidden by one of the initially selected peaks, or an irrelevant higher-energy configuration. In order to find and tag all locally stable configurations, one can discard all product intervals the minimum energy configuration of which is above a baseline energy and above which no peaks were selected at the beginning. In order to better visualize how configurations relate to each other and better resolve peaks by dimension, we instead split our data into the intervals defined over distance first and marked product interval minima in plots over the remaining two dimensions in Sect. 2.4.

## Supplementary Information

Supplementary Information 1.

## References

[1] Lee Y (2020). Universal oriented van der Waals epitaxy of 1d cyanide chains on hexagonal 2d crystals. Adv. Sci..

[2] Riede M, Mueller T, Tress W, Schueppel R, Leo K (2008). Small-molecule solar cells—status and perspectives. Nanotechnology.

[3] Meyer J (2011). Molecules for organic electronics studied one by one. Phys. Chem. Chem. Phys..

[4] Chu Y, Qian C, Chahal P, Cao C (2019). Printed diodes: Materials processing, fabrication, and applications. Adv. Sci..

[5] Zhu Y (2018). Deepscreen: An accurate, rapid, and anti-interference screening approach for nanoformulated medication by deep learning. Adv. Sci..

[6] Himanen L, Geurts A, Foster AS, Rinke P (2019). Data-driven materials science: Status, challenges, and perspectives. Adv. Sci..

[7] Aradi B, Hourahine B, Frauenheim T (2007). Dftb+, a sparse matrix-based implementation of the dftb method. J. Phys. Chem. A.

[8] Montoya JH, Persson KA (2017). A high-throughput framework for determining adsorption energies on solid surfaces. NPJ Comput. Mater..

[9] Blatov VA (2021). High-throughput systematic topological generation of low-energy carbon allotropes. NPJ Comput. Mater..

[10] Horton MK, Montoya JH, Liu M, Persson KA (2019). High-throughput prediction of the ground-state collinear magnetic order of inorganic materials using density functional theory. NPJ Comput. Mater..

[11] Lenz M-O (2019). Parametrically constrained geometry relaxations for high-throughput materials science. NPJ Comput. Mater..

[12] Zhang Y (2019). High-throughput 3d reconstruction of stochastic heterogeneous microstructures in energy storage materials. NPJ Comput. Mater..

[13] Kabiraj A, Kumar M, Mahapatra S (2020). High-throughput discovery of high curie point two-dimensional ferromagnetic materials. NPJ Comput. Mater..

[14] Torelli D, Moustafa H, Jacobsen KW, Olsen T (2020). High-throughput computational screening for two-dimensional magnetic materials based on experimental databases of three-dimensional compounds. NPJ Comput. Mater..

[15] Sauceda D (2021). High-throughput reaction engineering to assess the oxidation stability of max phases. NPJ Comput. Mater..

[16] Choudhary K (2020). High-throughput density functional perturbation theory and machine learning predictions of infrared, piezoelectric, and dielectric responses. NPJ Comput. Mater..

[17] Wang Z (2021). Machine learning method for tight-binding hamiltonian parameterization from ab-initio band structure. NPJ Comput. Mater..

[18] Vitale V (2020). Automated high-throughput wannierisation. NPJ Comput. Mater..

[19] Yu M, Yang S, Wu C, Marom N (2020). Machine learning the hubbard u parameter in dft+u using bayesian optimization. NPJ Comput. Mater..

[20] Zunger A, Wei S-H, Ferreira LG, Bernard JE (1990). Special quasirandom structures. Phys. Rev. Lett..

[21] Oses C, Toher C, Curtarolo S (2020). High-entropy ceramics. Nat. Rev. Mater..

[22] Addicoat MA, Coupry DE, Heine T (2014). Autografs: Automatic topological generator for framework structures. J. Phys. Chem. A.

[23] Yang S (2020). Ogre: A python package for molecular crystal surface generation with applications to surface energy and crystal habit prediction. J. Chem. Phys..

[24] Curtarolo S (2012). Aflow: An automatic framework for high-throughput materials discovery. Comput. Mater. Sci..

[25] Lokamani (2021). A combined experimental and theoretical study of 1,4-bis(phenylethynyl)-2,5-bis(ethoxy)benzene adsorption on Au(111). Surf. Sci..

[26] Ferrenberg AM, Landau DP, Binder K (1991). Statistical and systematic errors in Monte Carlo sampling. J. Stat. Phys..

[27] Landau DP (1976). Finite-size behavior of the ising square lattice. Phys. Rev. B.

[28] Tokdar ST, Kass RE (2010). Importance sampling: A review. WIREs Comput. Stat..

[29] Kunze T, Gemming S, Numazawa S, Schreiber M (2010). Low-temperature modeling for degenerate and frustrated heisenberg systems with anisotropy. Comput. Phys. Commun..

[30] Böhringer M (1999). Two-dimensional self-assembly of supramolecular clusters and chains. Phys. Rev. Lett..

[31] Meyer J (2015). Tuning the formation of discrete coordination nanostructures. Chem. Commun..

[32] Robles R (2020). Supramolecular chemistry based on 4-acetylbiphenyl on au(111). Phys. Chem. Chem. Phys..

[33] Kühne T (2020). Stm induced manipulation of azulene-based molecules and nanostructures: The role of the dipole moment. Nanoscale.

[34] Fihey A (2011). Scc-dftb parameters for simulating hybrid gold-thiolates compounds. J. Comput. Chem..

[35] Richard RM, Herbert JM (2011). Time-dependent density-functional description of the 1la state in polycyclic aromatic hydrocarbons: Charge-transfer character in disguise?. J. Chem. Theory Comput..

[36] Elstner M (1998). Self-consistent-charge density-functional tight-binding method for simulations of complex materials properties. Phys. Rev. B.

[37] Niehaus T, Elstner M, Frauenheim T, Suhai S (2001). Application of an approximate density-functional method to sulfur containing compounds. J. Mol. Struct. Theochem..

[38] Rappe AK, Casewit CJ, Colwell KS, Goddard WA, Skiff WM (1992). Uff, a full periodic table force field for molecular mechanics and molecular dynamics simulations. J. Am. Chem. Soc..

