# Supplementary material for: Describing chain-like assembly of ethoxygroup-functionalized organic molecules on Au(111) using high-throughput simulations
Source: Sci Rep. 2021 Jul 19;11:14649. doi: 10.1038/s41598-021-93724-5 (PMC8290052; doi:10.1038/s41598-021-93724-5)
Supplement: Supplementary file 1 — Supplementary Information 1. [file 41598_2021_93724_MOESM1_ESM.pdf]

Describing chain-like assembly of ethoxygroup-functionalized organic molecules on Au(111) using high-throughput simulations

Lokamani(1,2,3), Jeffrey Kelling(1), Robin Ohmann(3,4), Jörg Meyer(3), Tim Kühne(5), Gianaurelio Cuniberti(3), Jannic Wolf(6), Guido Juckeland(1), Thomas Huhn(6), Peter Zahn(2), Francesca Moresco(5) and Sibylle Gemming(5,7)

(1)Department of Information Services and Computing,
Helmholtz-Zentrum Dresden-Rossendorf (HZDR),
Bautzner Landstraße 400, 01328 Dresden, Germany
(2)Institute of Ion Beam Physics and Materials Research,
Helmholtz-Zentrum Dresden-Rossendorf (HZDR),
Bautzner Landstraße 400, 01328 Dresden, Germany
(3)Institute for Materials Science,
Technische Universität Dresden, 01062 Dresden, Germany
(4)Department Physik, Universität Siegen,
Walter-Flex-Straße 3, 57072 Siegen, Germany
(5)Center for Advancing Electronics Dresden,
Technische Universität Dresden, 01062 Dresden, Germany
(6)Department of Chemistry,
Universität Konstanz, 78457 Konstanz, Germany
(7)Institute of Physics, Technische Universität Chemnitz, 09107 Chemnitz, Germany

I. ONE-DIMENSIONAL ROW-LIKE STRUCTURES AT HIGH COVERAGE

At high coverage, 1,4-bis(phenylethynyl)-2,5-bis(ethoxy)benzene (PEEB) molecules form one-dimensional row-like structures. The row-like coverage can be considered as repetition of *normal*-fence structures, as depicted using overlay of schematics of PEEB and STM image. Shifts in the row-like structures, marked with red arrows, can be attributed to change in chirality of the PEEB molecules.

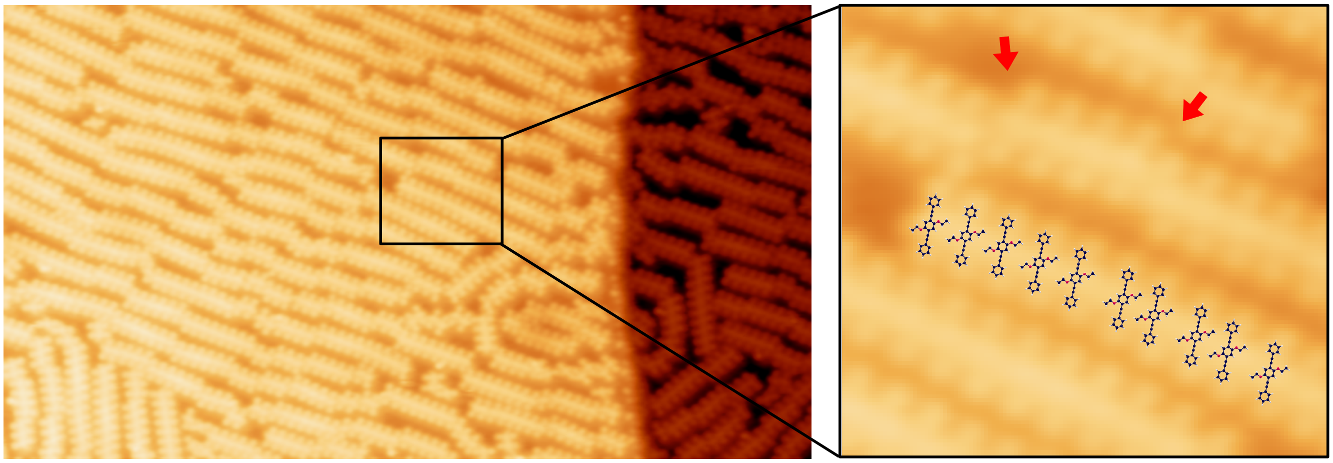

(a) Mono-layer (b) row formation Normal fence

Figure S1. (a) STM image of PEEB molecules at high coverage on Au(111) (b) Zoom-in of a row-structure with schematics of PEEB lined up together. Image dimensions: $70 \times 40\,\text{nm2}$. STM Parameters: $1\,\text{V}$ and $0.05\,\text{nA}$.

II. SAMPLING

Periodic boundary conditions imposed in supercell calculations with one molecule per supercell induce additional interactions between periodic images of the molecules. We model the Au(111) surface by a supercell that laterally has the dimension 10×10 atoms [28.85 Å × 28.85 Å]. The short and long diagonals have lengths of 28.85 Å and 49.97 Å, respectively. Spurious interactions between the molecule and its periodic images are most likely along the short diagonal, where the shortest distance between neighbouring cells occurs. One such scenario for the largest possible molecule-molecule interaction is shown for $\beta = 90°$. The shortest distance possible between the molecule and its periodic image is 10.4 Å, which corresponds to the distance between the peripheral hydrogen atoms as indicated by the double-headed arrow. The distance between centers of mass of the molecule and its periodic image is 28.85 Å. Considering the fact, that the cutoff used in the tight-binding parameter set from Ref. [1] for C, H and O atoms is 5 Å, the interactions between the periodic images may be neglected.

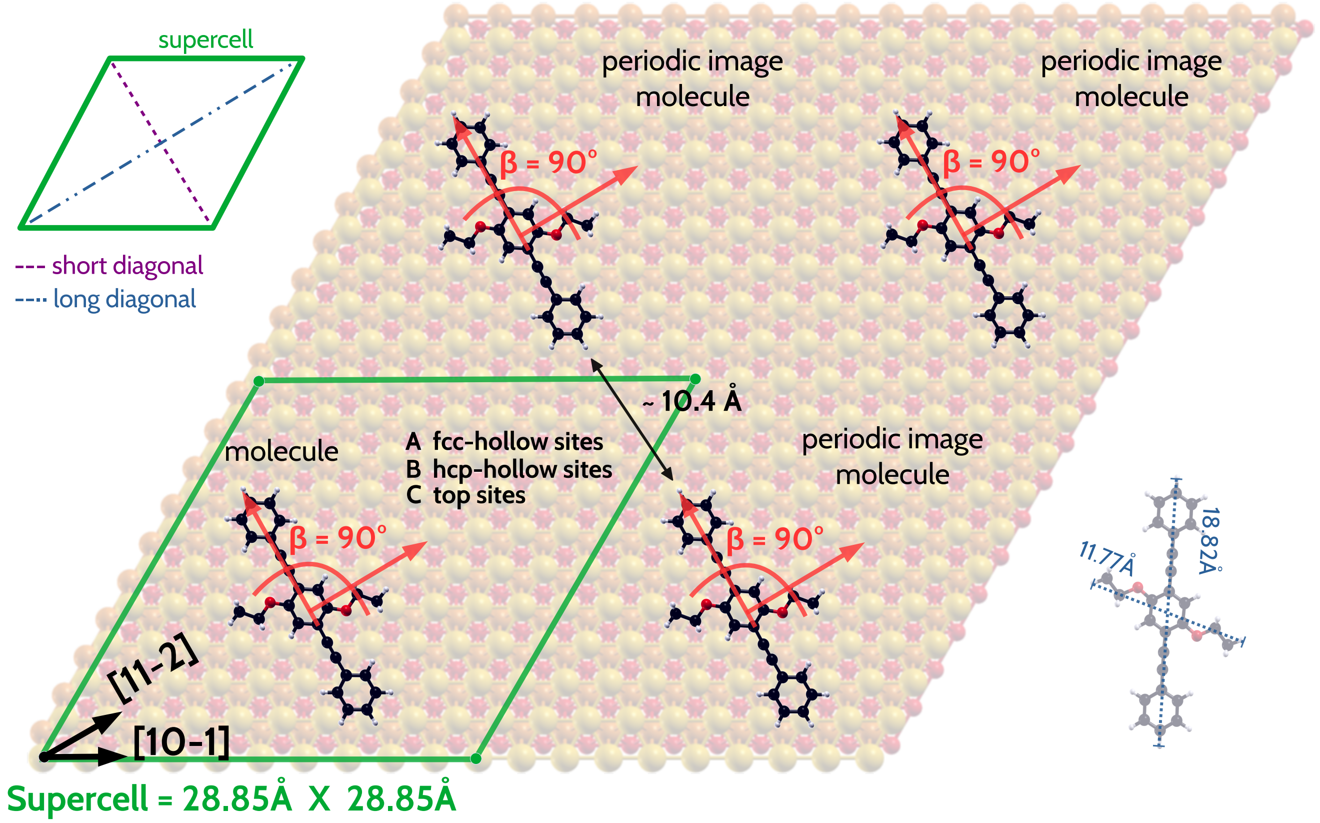

Figure S2. Supercell geometry employed to calculate the interaction energy between a single PEEB molecule and the Au(111) surface obeying the minimum image convention.

Periodic boundary conditions imposed during clustering simulations induce additional interactions between periodic images of molecule pairs. The supercell used has the dimension 20 × 20 Au atoms [57.7 Å × 57.7 Å], corresponding to 20×20 Au atoms. The short and long diagonals have a length of 57.7 Å and 99.94 Å, respectively. Spurious interactions between the first/second molecule and the periodic image of the second/first molecule are most likely along the short diagonal. One such scenario for a maximum possible spurious interaction is shown for $\beta_1 = \beta_2 = 90°$. Since we are interested only in the short-range order, interaction within a pair of molecules up to a maximum distance of 22 Å (shown using grey dotted line) are considered. The minimal possible distance between first/second molecule and the periodic image of the second/first molecule turns out to be around 17 Å, as shown using a double-headed arrow. This interaction is between peripheral hydrogen atoms. Since the cutoff used in parameter set from Ref. [1] for C, H and O atoms is 5 Å, the interactions due to the periodic images are negligibly small and can be safely neglected.

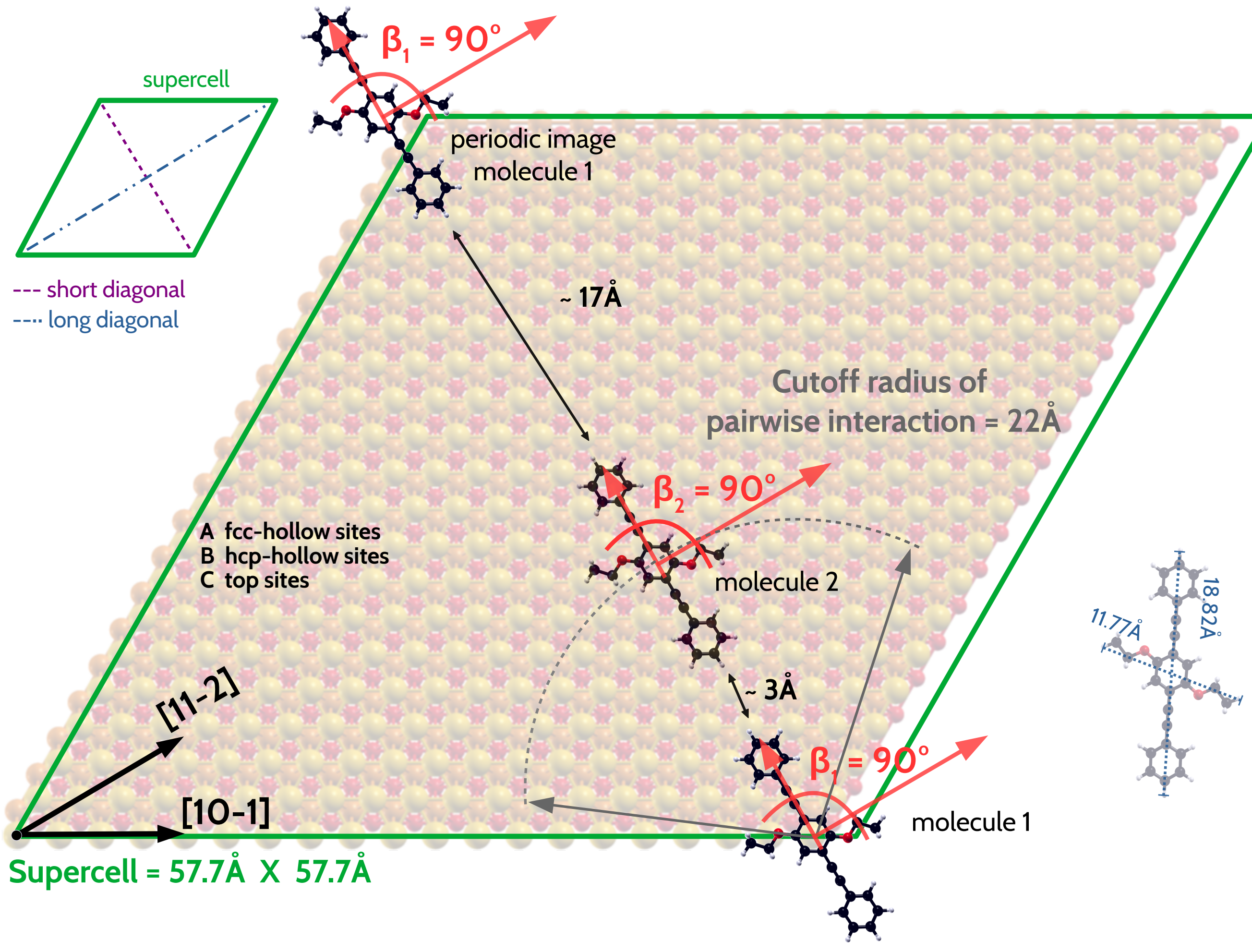

Figure S3. Spurious interactions in simulations modelling pairwise interactions

III. SELECTED CONFIGURATIONS

Here we present selected molecular pairs, which arise from the selection procedure based on energy function and peak structure in dependence on R_{12}, α and $\Delta\beta$.

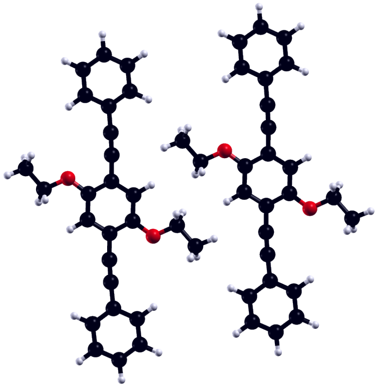

(# 1.1): $\alpha = 78.0°, \beta_1 = 45.0°, \Delta\beta = 0.0°, R_{12} = 8.6550$ Å

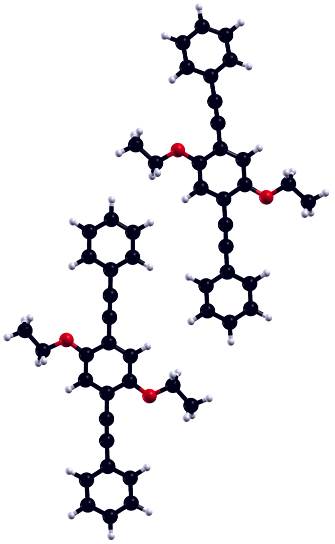

(# 2.1): $\alpha = 33.7°, \beta_1 = 168.0°, \Delta\beta = 6.0°, R_{12} = 12.5020$ Å

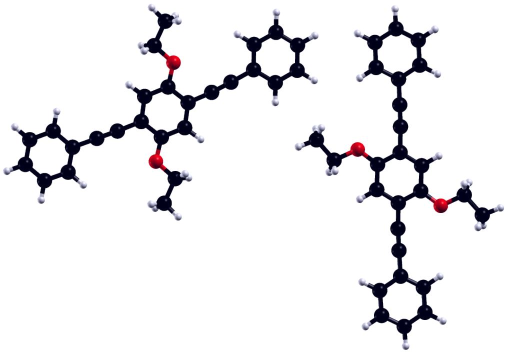

(# 2.2): $\alpha = 38.0°, \beta_1 = 183.0°, \Delta\beta = 114.0°, R_{12} = 13.7180$ Å

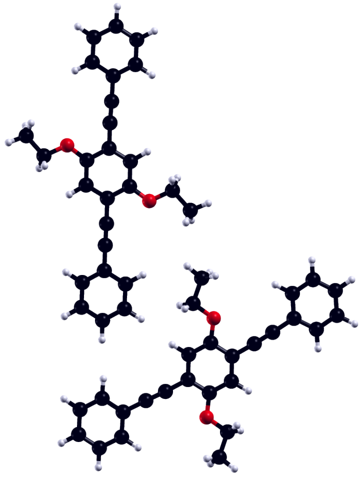

(# 2.4): $\alpha = 90.4°, \beta_1 = 108.0°, \Delta\beta = 60.0°, R_{12} = 12.2644$ Å

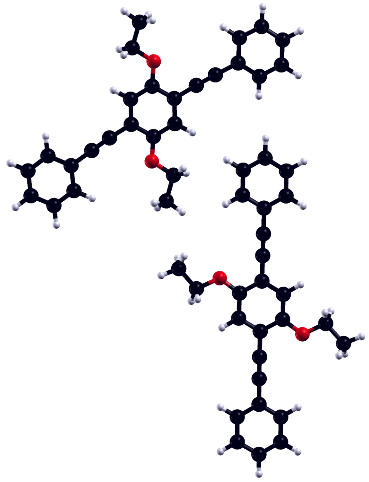

(# 2.5): $\alpha = 91.8°, \beta_1 = 132.0°, \Delta\beta = 123.0°, R_{12} = 12.3597\,\text{Å}$

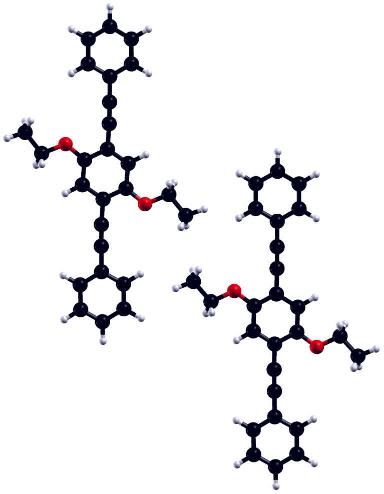

(# 2.7): $\alpha = 125.4°, \beta_1 = 69.0°, \Delta\beta = 3.0°, R_{12} = 12.5789\,\text{Å}$

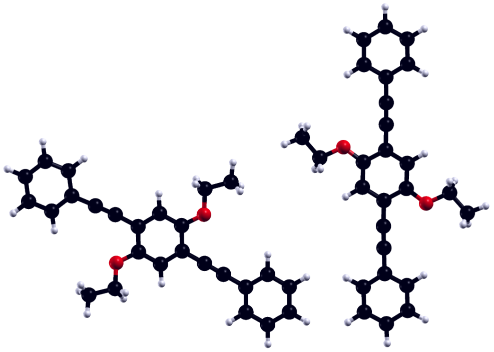

(# 2.8): $\alpha = 134.8°, \beta_1 = 183.0°, \Delta\beta = 63.0°, R_{12} = 13.1733\,\text{Å}$

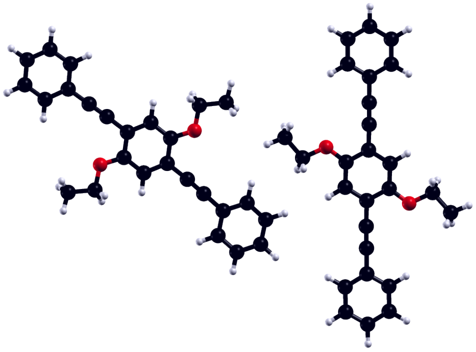

(# 2.10): $\alpha = 148.7°, \beta_1 = 126.0°, \Delta\beta = 54.0°, R_{12} = 12.4813\,\text{Å}$

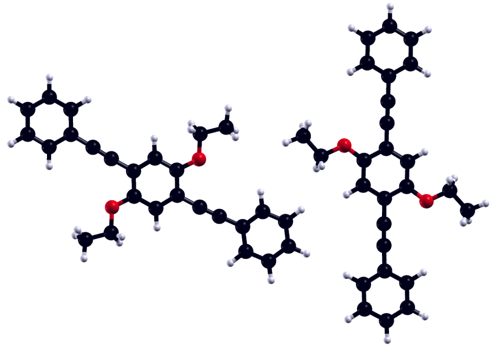

(# 2.11): $\alpha = 149.0°, \beta_1 = 48.0°, \Delta\beta = 117.0°, R_{12} = 12.9779\,\text{Å}$

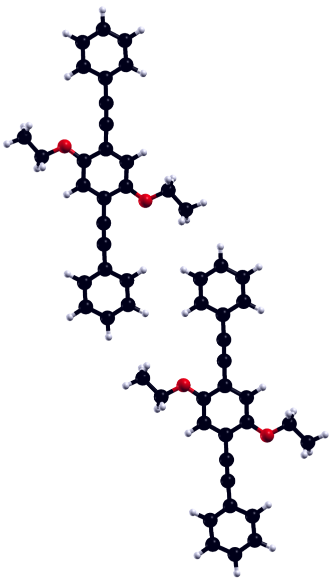

(# 3.1): $\alpha = 153.9°, \beta_1 = 162.0°, \Delta\beta = 3.0°, R_{12} = 15.2883\,\text{Å}$

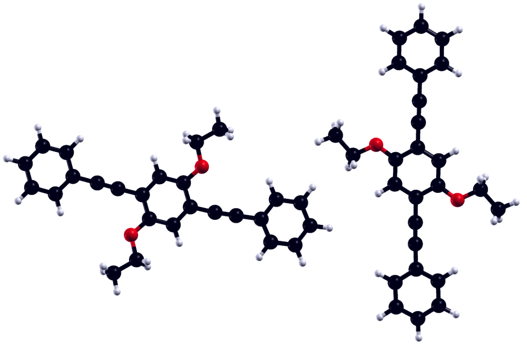

(# 3.2): $\alpha = 160.0°, \beta_1 = 63.0°, \Delta\beta = 102.0°, R_{12} = 14.3716\,\text{Å}$

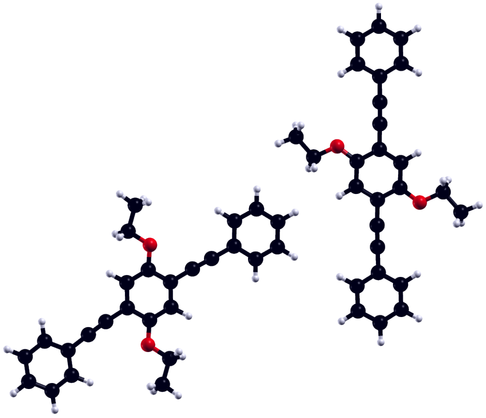

(# 3.4): $\alpha = 2.7°, \beta_1 = 63.0°, \Delta\beta = 57.0°, R_{12} = 14.5445\,\text{Å}$

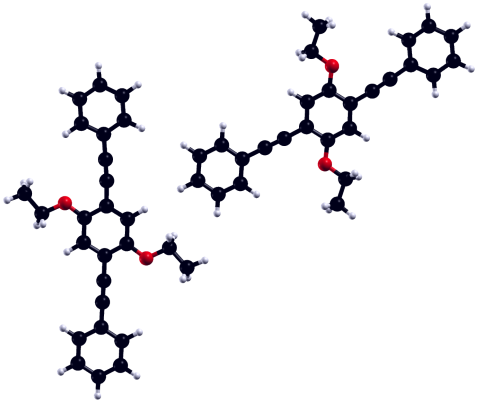

(# 3.5): $\alpha = 0.2°, \beta_1 = 123.0°, \Delta\beta = 120.0°, R_{12} = 14.4023\,\text{Å}$

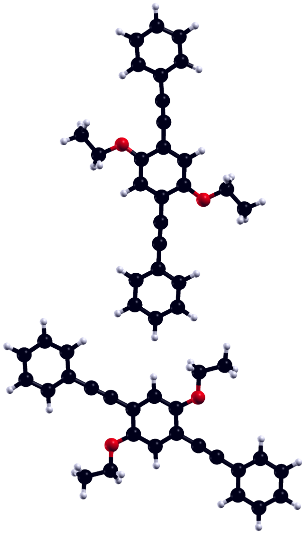

(# 3.7): $\alpha = 62.4°, \beta_1 = 6.0°, \Delta\beta = 117.0°, R_{12} = 14.2514\,\text{Å}$

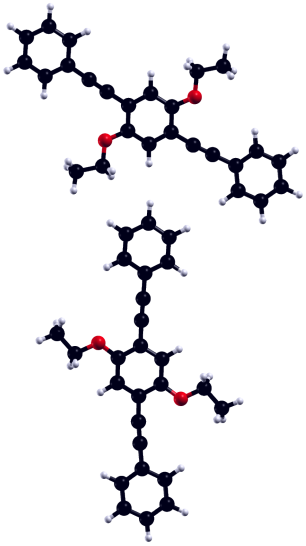

(# 3.8): $\alpha = 59.9°, \beta_1 = 123.0°, \Delta\beta = 60.0°, R_{12} = 14.3077\,\text{Å}$

[1] M. Elstner, D. Porezag, G. Jungnickel, J. Elsner, M. Haugk, T. Frauenheim, S. Suhai, and G. Seifert, Self-consistent-charge density-functional tight-binding method for simulations of complex materials properties, Phys. Rev. B **58**, 7260 (1998).
